# Supplementary material for: Dual targeting of MAPK and PI3K pathways unlocks redifferentiation of Braf-mutated thyroid cancer organoids
Source: Oncogene. 2023 Nov 20;43(3):155–70. doi: 10.1038/s41388-023-02889-y (PMC10786723; doi:10.1038/s41388-023-02889-y)
Supplement: Supplementary file 1 — Supplementary material [file 41388_2023_2889_MOESM1_ESM.pdf]

SUPPLEMENTARY MATERIAL

Supplementary figures

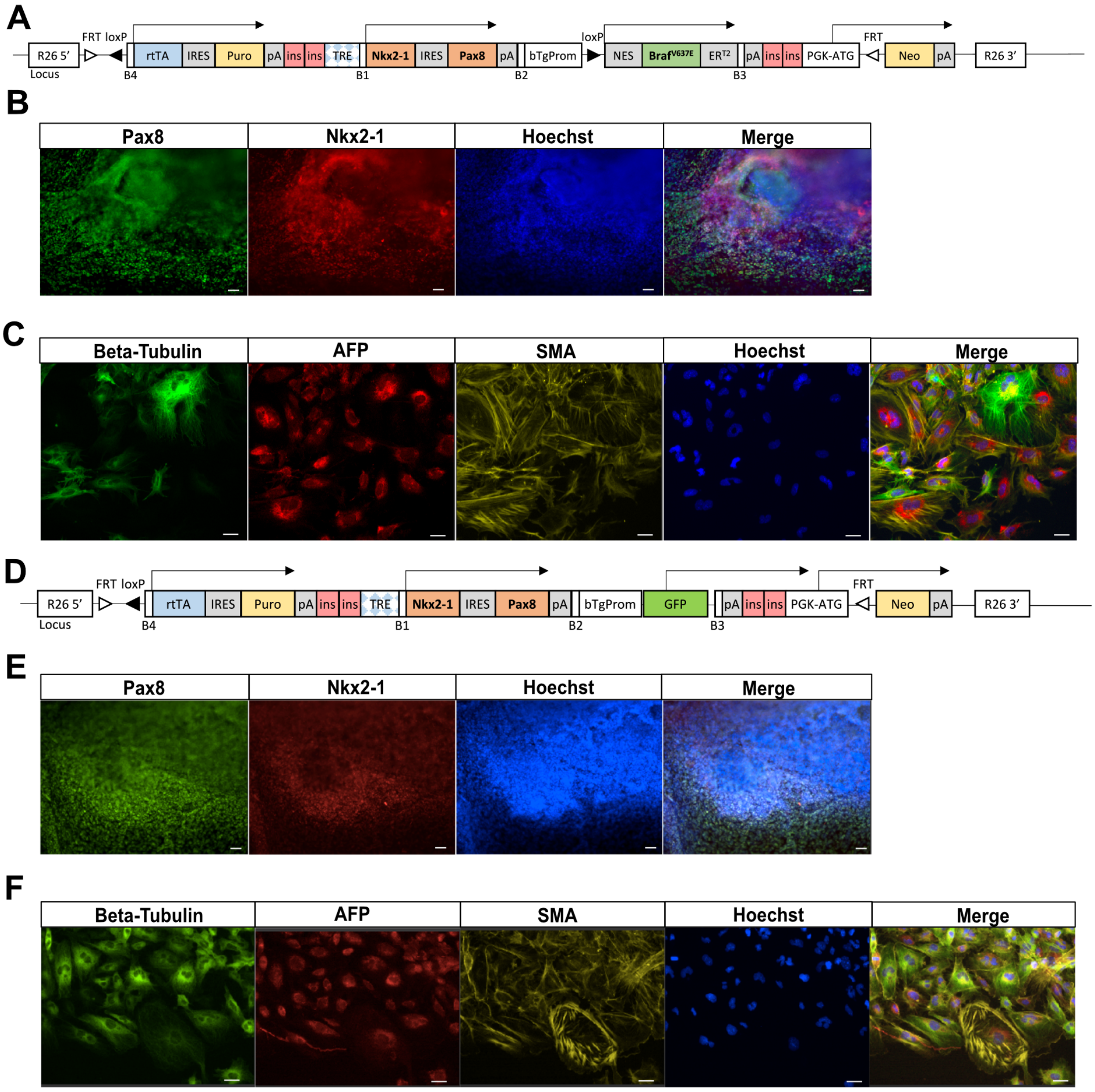

Supplementary Fig. 1 mESC-cell lines generation and characterization. Schematic representation of the mESC_Nkx2-1/Pax8_bTg_Braf^{V637E} cell line (A). mESC_Nkx2-1/Pax8_bTg_Braf^{V637E} line response to doxycycline treatment shows expression of Pax8 and Nkx2-1 proteins (B). Spontaneous differentiation mESC_Nkx2-1/Pax8_bTg_Braf^{V637E} cell line leads to the generation of ectodermal (Beta-Tubulin), endodermal (AFP) and mesodermal (SMA) cells (C). Schematic representation of the mESC_Nkx2-1/Pax8_bTg_eGFP control cell line (D). Dox treatment leads to an increase

in Pax8 and Nkx2-1 expression among control cells (E). Spontaneous differentiation of the control cells leads to the generation of ectodermal (Beta-Tubulin), endodermal (AFP) and mesodermal (SMA) cells (F). Scale bars, 50 µm (B, E) and 10 µm (C, F).

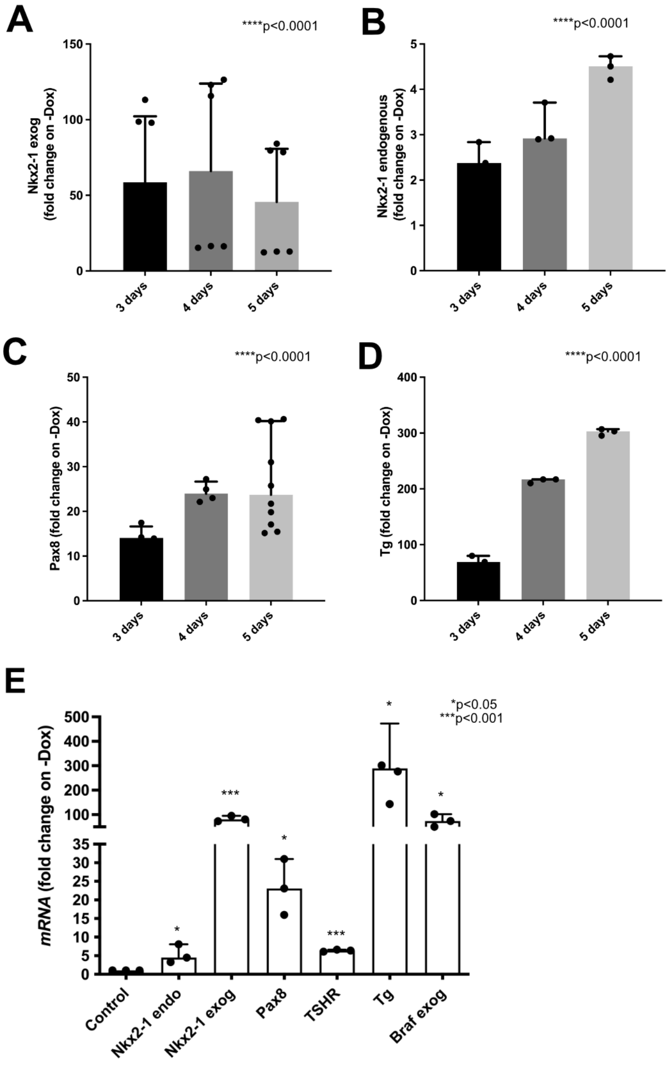

Supplementary Fig. 2 Doxycycline treatment for 5 days leads to more efficient thyroid gene expression induction. Time course of Dox-dependent induction of exogenous and endogenous *Nkx2-1* (A, B) *Pax8* (C) and *Tg* (D) expression. mRNA levels of thyroid genes at day 9, after 5 days of Dox treatment (E). Bar graphs represent the median (IQR) of at least 3 independent experiments with individual values shown.

Comparisons were performed against the -Dox condition. (*p<0.05 ; **p<0.01 ; ***p<0.001; ***p<0.0001; Kruskal-Wallis test (A-D) and Mann-Whitney U test (E)).

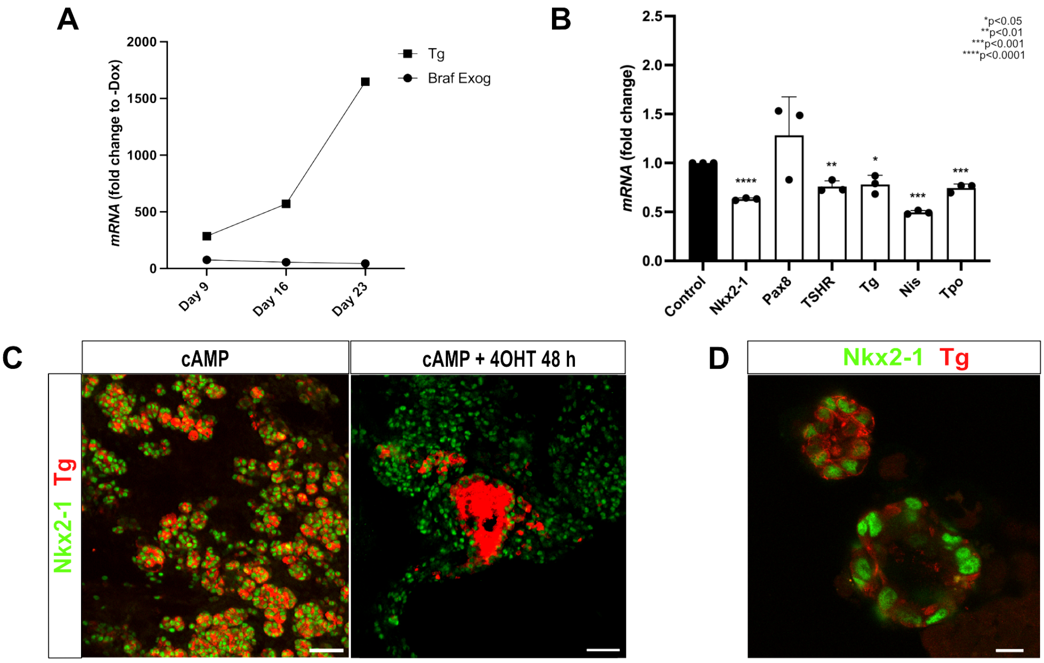

Supplementary Fig. 3 4OHT treatment rapidly induces Braf-carcinogenic changes in gene expression and follicular organization. Endogenous Tg and exogenous Braf expression levels at day 9, 16, and 23 of thyroid differentiation protocol (A). Induction of Braf^{V637E} for 8 hours promotes the downregulation of main thyroid genes (B). Bar graphs represent the median (IQR) of 3 independent experiments with individual values shown. Comparisons were performed against the cAMP condition. (*p<0.05 ; **p<0.01 ; ***p<0.001; ***p<0.0001; Mann-Whitney U test). Nkx2-1 and Tg immunostaining shows that under Braf^{V637E} activation by 4OHT (48 h) thyroid follicles present a bigger size and heterogeneous distribution of Tg compared to the control (cAMP) (C). Remaining follicular-like structures after Braf^{V637E} activation (4OHT; 48 h) (D). Scale bars, 50 μm (C) and 10 μm (D).

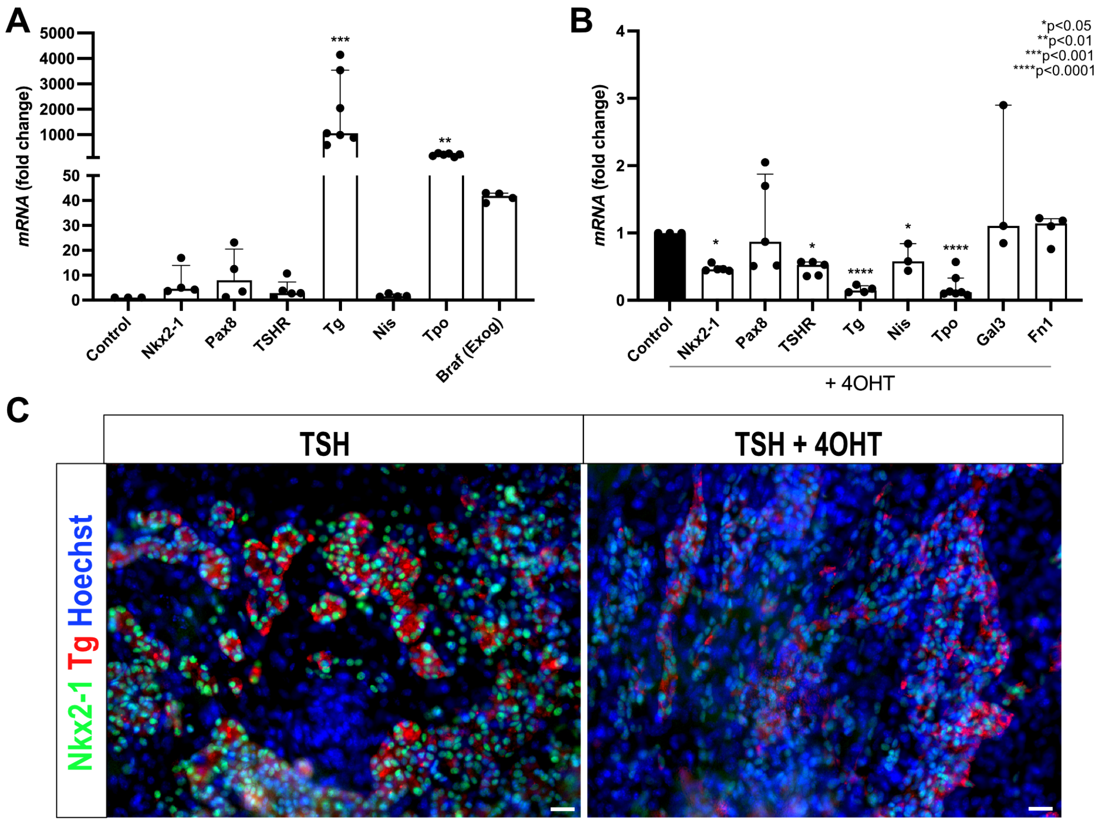

Supplementary Fig. 4 Braf^{V637E} activation in TSH-differentiated thyroid organoids lead to similar phenotype than cAMP-treated cancer organoids. Thyroid genes expression levels ^{were} obtained when mESC_Nkx2-1/Pax8_bTg_Braf^{V637E}_ERT^{2} cells were differentiated using hrTSH (A). Treatment with 4OHT for 7 days led to downregulation of thyroid maturation genes, *TSHR, Tg, Nis* and *Tpo* when compared to cAMP control organoids (B). Bar graphs represent the median (IQR) of 3 independent experiments with individual values shown. Comparisons were performed against the cAMP condition. (*p<0.05 ; **p<0.01 ; ***p<0.001; ***p<0.0001; Mann-Whitney U test). Nkx2-1 and Tg immunostaining shows that under Braf^{V637E} activation by 4OHT (7 days) thyroid organoids lose the follicular organization while the levels of Tg protein is reduced compared to the cAMP control condition (C). Scale bars, 20 µm.

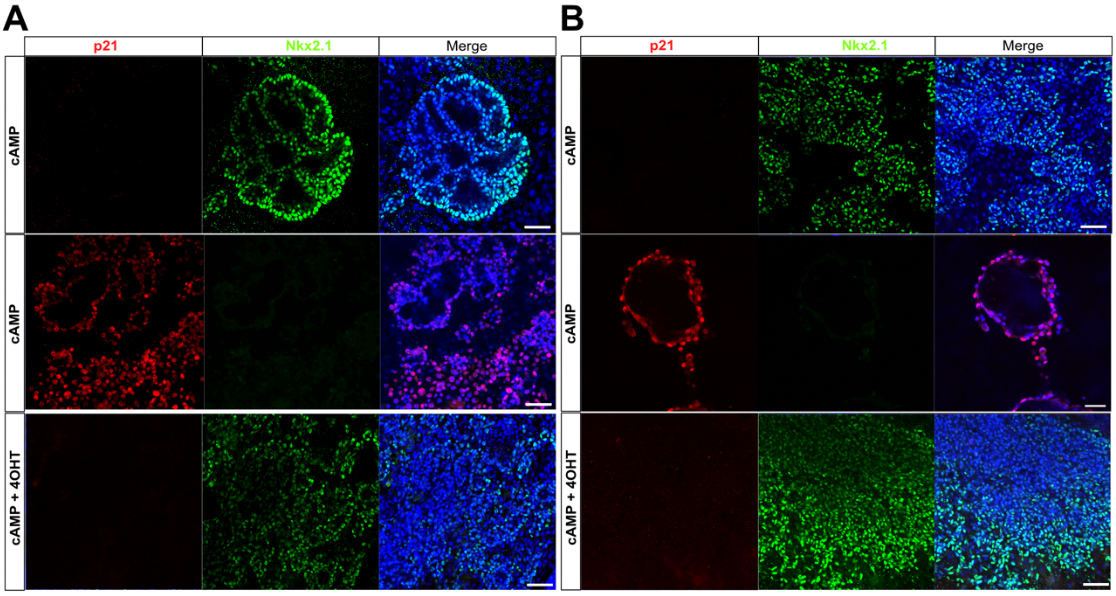

Supplementary Fig. 5 Braf^{V637E} induction does not induce the expression of p21 in thyroid organoids. Confocal images show the expression of Nkx2.1 and p21 after 7 (A) and 21 days (B) after induction of *Braf* oncogene by 4OHT treatment. The absence of co-expression of p21 among the Nkx2.1 thyroid cells indicates the absence of oncogene-induced senescence among the cancer organoids. Scale bars, 50 µm.

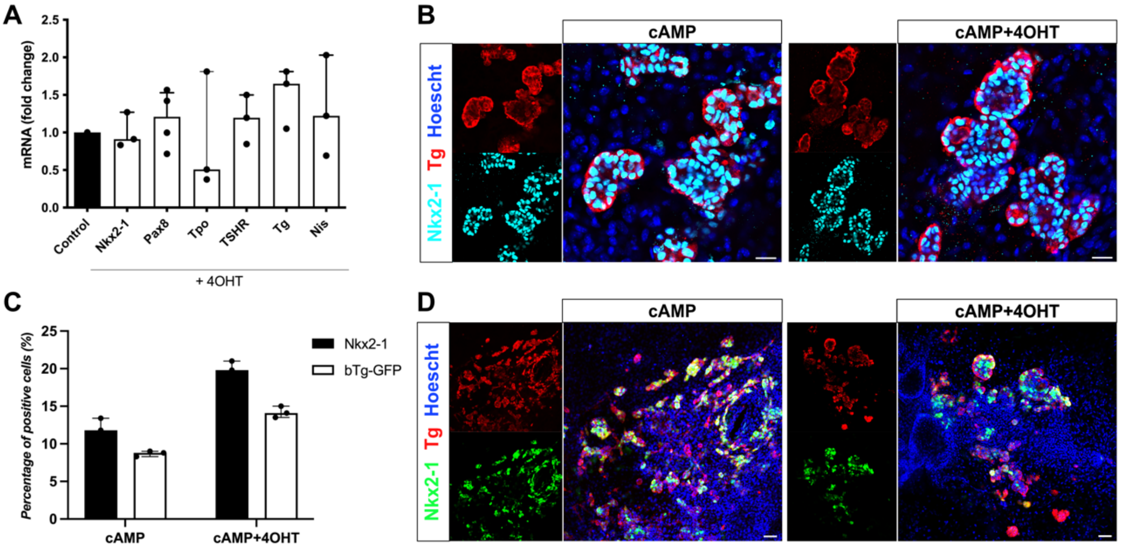

Supplementary Fig. 6 Treatment with 4OHT in WT thyroid organoids did not lead to cancer phenotype. TRE-Nkx2-1-Pax8_bTg-eGFP (WT) cell line treated for 7 days with 4OHT did not show changes in gene expression when compared to the control

(cAMP) condition (A). Bar graphs represent the median (IQR) of 3 independent experiments with individual values shown. Comparisons were performed against the cAMP (control) condition. (Mann-Whitney U test). Confocal images show that Tg and Nkx2-1 staining and the follicular structures are preserved after 7 days of 4OHT treatment (B). Quantification of the Nkx2-1 and GFP proportions among cAMP and 4OHT-treated (7 days) samples (C). Bar graphs represent the median (IQR) of 3 independent experiments with individual values shown. Comparisons were performed against the cAMP (control) condition. (Mann-Whitney U test). Confocal images show that Tg and GFP co-staining is found only in a proportion of the Tg-expressing cells (D). Scale bars, 50 μm.

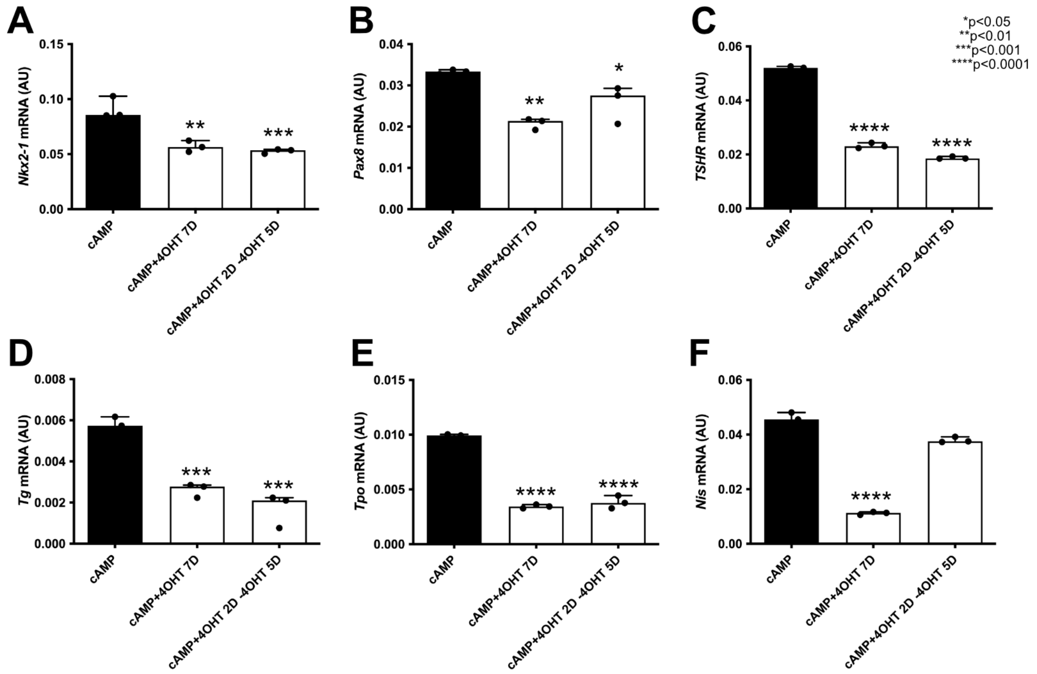

Supplementary Fig. 7 4OHT interruption does not restore the thyroid gene expression levels. The induction of Braf^{V637E} for 2 days followed by 4OHT removal for 5 days was not enough to recover *Nkx2-1* (A), *Pax8* (B), *TSHR* (C), *Tg* (D), *Tpo* (E) mRNA to the control levels (cAMP). However, it increased *Nis* expression (F) to control values (cAMP). Bar graphs represent the median (IQR) of 3 independent experiments

with individual values shown. Comparisons were performed against the cAMP condition. (*p<0.05 ; **p<0.01 ; ***p<0.001; ***p<0.0001; Mann-Whitney U test).

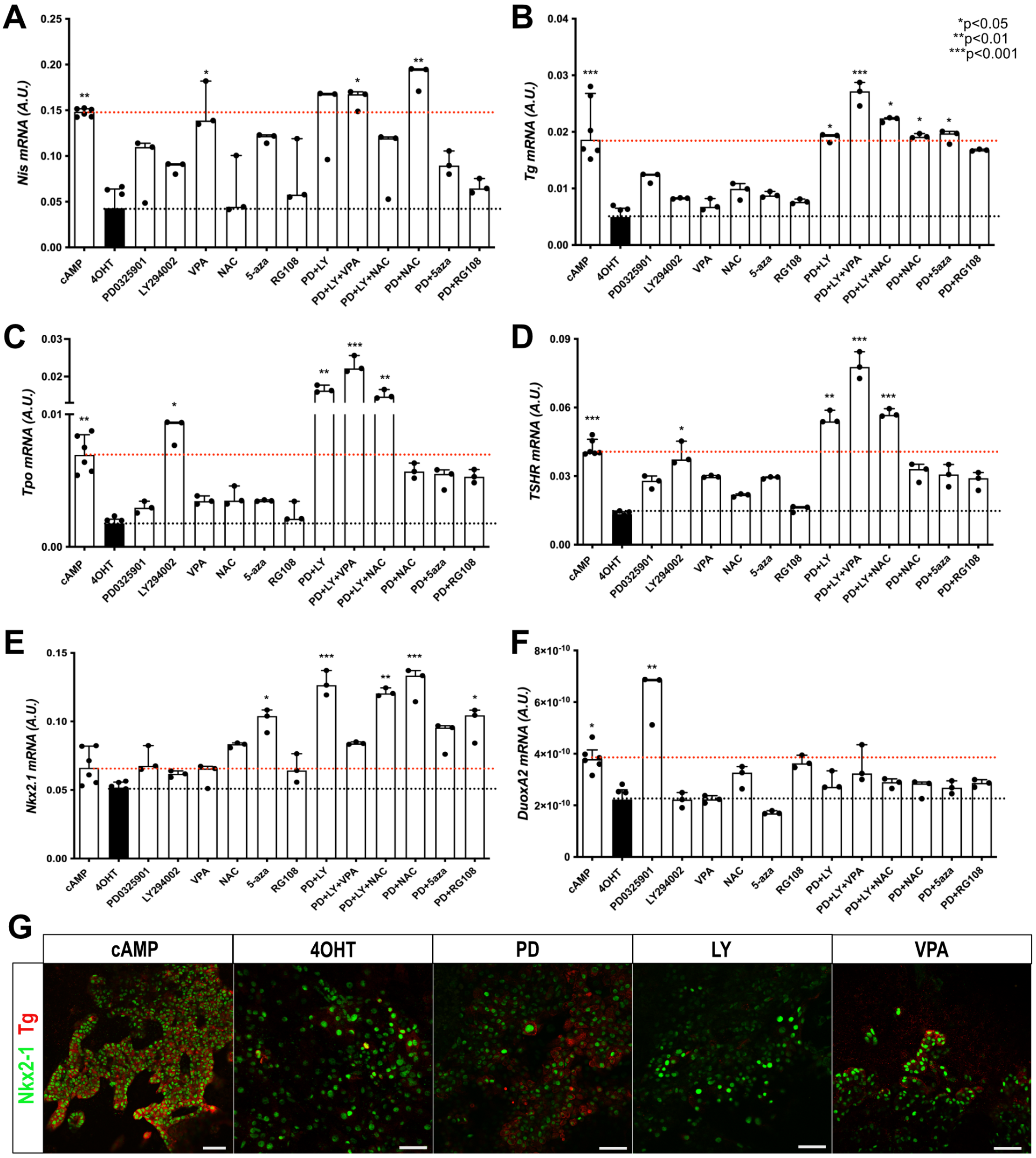

Supplementary Fig. 8 Effect of distinct classes of inhibitors on thyroid markers. Gene expression levels of *Nis* (A), *Tg* (B), *Tpo* (C), *TSHR* (D), *Nkx2.1* (E) *and Duox2A* (F) among normal thyroid organoids, Braf^{V637E}-expressing (4OHT) and treated with distinct inhibitor (4OHT+inhib). Bar graphs represent the median (IQR) of 3 independent experiments with individual values shown. Comparisons were performed against the 4OHT condition. (*p<0.05 ; **p<0.01 ; ***p<0.001; Mann-Whitney U test). Nkx2-1 and

Tg co-staining shows that isolated PD0325901, LY294002 and VPA treatments are insufficient to revert the inhibitory effect of Braf^{V637E} activation on Tg expression and thyroid follicular organization. Scale bar, 50 µm.

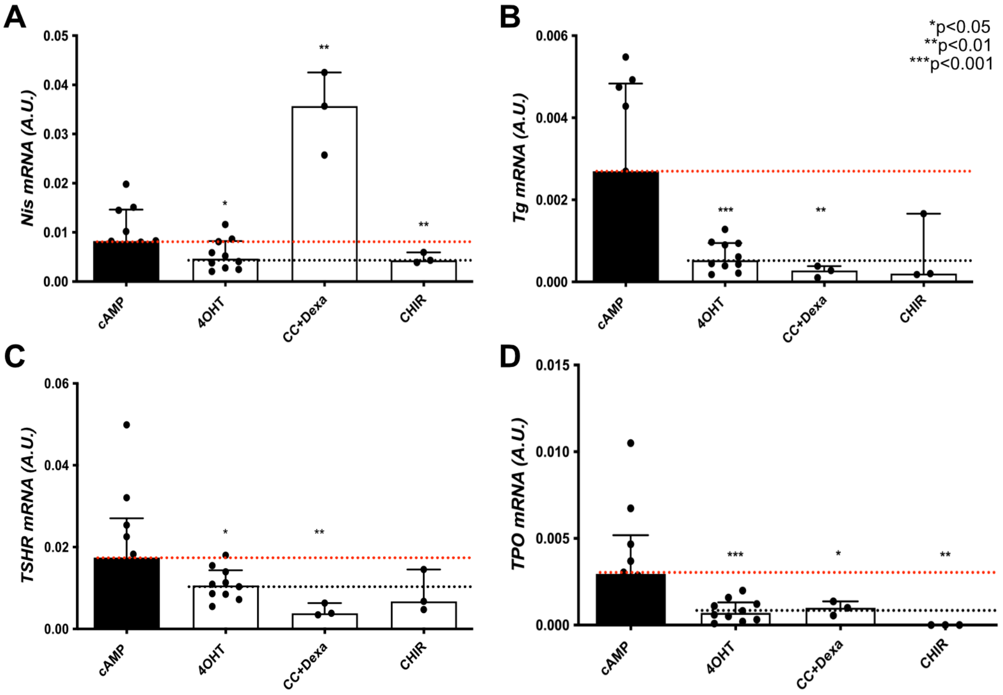

Supplementary Fig. 9 Inhibition of inflammation and induction of Wnt/β-catenin pathway are not enough to revert thyroid dedifferentiation. Treatment of cancer organoids (previously treated with 4OHT) with combined anti-inflammatory drugs, dexamethasone, and CC-5013 (TNF-alpha inhibitor) recovered *Nis* mRNA to control (cAMP) levels (A). On the other hand, nor dexamethasone+CC-5013, nor CHIR- 99021 (Wnt/β-catenin activator) could improve the expression levels of *Tg* (B), *TSHR* (C) and *Tpo* (D). Bar graphs represent the median (IQR) of 3 independent experiments with individual values shown. Comparisons were performed against the cAMP condition. (*p<0.05 ; **p<0.01 ; ***p<0.001; Mann-Whitney U test).

Supplementary tables

Supplementary Table 1. Antibodies information.

Primary antibodies						
Protein Target	***Provider***	***Catalog number***	***Host Species***	***Dilution Flow Cyt.***	***Dilution IF***	***Dilution WB***
AFP	Santa Cruz	sc-8108	Goat		1:100	
β-III Tubulin	Eurogentec	MMS-435P-200	Mouse		1:1,000	
αSMA	Abcam	ab32575	Rabbit		1:1,000	
Nkx2-1	Abcam	ab76013	Rabbit	1 :100	1:500	
Pax8	Cell Signaling	59019	Rabbit		1:500	
Tg	Dako	A0251	Rabbit		1:2,000	
Tg	Abcam	Ab187378	Mouse		1:250	
Nis	Gift from N. Carrasco		Rabbit		1:1,000	
Tg-I	Gift from C. Ris-Stalpers		Mouse		1:250	
E-cadherin	BD Biosciences	610181	Mouse		1:1,000	
ZO-1	Invitrogen	339100	Mouse		1:500	
Hoechst	Invitrogen	33342			1:1,000	
p-ERK1/2	Cell Signaling	4370	Rabbit			1:1,000
ERK1/2	Santa Cruz Biotechnology	sc-514302	Mouse			1 :400
β-Actin	Cell Signaling	3700	Mouse			1:1,000
FITC Rabbit Anti- Active Caspase-3	BD Biosciences	559341	Rabbit	1 :5		
APC anti-BrdU	BD Biosciences	552598		1 :50		
**Secondary antibodies**						
Cy3-conjugated	Jackson Immunoresearch	715-165-150	Donkey anti-mouse IgG		1:500	

Cy3-conjugated	Jackson Immunoresearch	711-165-152	Donkey anti-rabbit IgG		1:500	
Cy3-conjugated	Jackson Immunoresearch	705-165-147	Donkey anti-goat IgG		1:500	
Alexa fluor 488-conjugated	Jackson Immunoresearch	715-545-150	Donkey anti-mouse IgG		1:500	
Alexa fluor 647-conjugated	Jackson Immunoresearch	715-605-150	Donkey anti-mouse IgG		1:500	
Alexa fluor 647-conjugated	Jackson Immunoresearch	711-605-152	Donkey anti-rabbit IgG	1:300	1:500	
Anti-rabbit IgG, HRP-linked	Cell Signaling	7074				1:1,000
Anti-mouse IgG, HRP-linked	Cell Signaling	7076				1:1,000

Supplementary Table 2. List of primers used for RT-qPCR analysis.

**Gene**	**Primer Forward**	**Primer Reverse**
β2m-Globulin	GCTTCAGTCGTCAGCATGG	CAGTTCAGTATGTTCGGCTTCC
Nkx2-1 exog.	GGCGCCATGTCTTGTTCT	ACACCGGCCTTATTCCAAG
Nkx2-1	GGCGCCATGTCTTGTTCT	GGGCTCAAGCGCATCTCA
Pax8	CAGCCTGCTGAGTTCTCCAT	CTGTCTCAGGCCAAGTCCTC
Foxe1	GGCGGCATCTACAAGTTCAT	GGATCTTGAGGAAGCAGTCG
Tg	GTCCAATGCCAAAATGATGGTC	GAGAGCATCGGTGCTGTTAAT
Nis/Slc5a5	AGCTGCCAACACTTCCAGAG	GATGAGAGCACCACAAAGCA
Tshr	GTCTGCCCAATATTTCCAGGATCTA	GCTCTGTCAAGGCATCAGGGT
Tpo	ACAGTCACAGTTCTCCACGGATG	ATCTCTATTGTTGCACGCCCC
$Braf^{V637E}$ exog.	GCTTGTGCTTCTCCGAAAAC	AAGGCCAGGCTGTTCTTCTT
Dio1	ATGGCCAGGAACCCCCG	GCCTGCTGCCTTGAATGAAA
Dio2	TTCTCCAACTGCCTCTTCCTG	CCCATCAGCGGTCTTCTCC

Duoxa2	GCCTGGAATCCGTGGCACTC	TCCCCACGAACCAGTCTCCACT
Gal3	TTGAAGCTGACCACTTCAAGGTT	AGGTTCTTCATCCGATGGTTGT
Fn1	AATCACAGTAGTTGCGGCAGGAGA	TCTGTCCCAGGCAGGAGATTTGTT

Supplementary Table 3. Gene list used for thyroid differentiation (TDS and eTDS) and ERK scores calculation.

	**TDS**	**eTDS**	**ERK score**
**Included**	DIO1<br>DIO2<br>DUOX1<br>DUOX2<br>FOXE1<br>GLIS3<br>NKX2-1<br>PAX8<br>SLC26A4<br>SLC5A5<br>SLC5A8<br>TG<br>THRA<br>THRB<br>TPO<br>TSHR	BCL2<br>CDH16<br>CLCNKB<br>DLG2<br>DPP6<br>FAM167A<br>FHL1<br>FLRT1<br>GPM6A<br>GRIN2C<br>HGD<br>KIT<br>LMOD1<br>LRP2<br>MATN2<br>MPPED2<br>PKHD1L1<br>PLA2R1<br>SLC26A7<br>SLC4A4<br>SORBS2<br>STXBP5L<br>TFCP2L1<br>TFF3<br>TPPP<br>WSCD2<br>ZMAT4	ARID5A<br>B4GALT6<br>BRIX1<br>BYSL<br>CCND1<br>CD3EAP<br>CHSY1<br>DDX21<br>DUSP4<br>DUSP6<br>EGR1<br>ELOVL6<br>ETV1<br>ETV4<br>ETV5<br>FOS<br>FOSL1<br>GEMIN4<br>GTF2A1L<br>GNL3<br>GPR3<br>GTPBP4<br>HMGA2<br>HYDIN<br>IER3<br>KIR3DL2<br>LIF<br>MAFF<br>MAP2K3<br>MYC<br>NOP16<br>PHLDA2<br>PLK3<br>POLR1C<br>POLR3G<br>PPAN<br>PPAT<br>PYCRL<br>RRS1<br>SEMA6A<br>SH2B3<br>SLC1A5<br>SLC4A7<br>SPRED2<br>SPRY2<br>SPRY4<br>TNC<br>TNFRSF12A<br>TSR1<br>WDR3<br>YRDC
**Missing**		LOC286002<br>MT1G	CXCL8

Supplementary Table 4. List of the DEGs for each gene enrichment classification.

Included as a separate file.
